# Temporomandibular Joint Osseous Morphology of Class I and Class II Malocclusions in the Normal Skeletal Pattern: A Cone-Beam Computed Tomography Study

**DOI:** 10.3390/diagnostics11030541

**Published:** 2021-03-18

**Authors:** Xiao-Chuan Fan, Lin-Sha Ma, Li Chen, Diwakar Singh, Xiaohui Rausch-Fan, Xiao-Feng Huang

**Affiliations:** 1 Department of Stomatology, Beijing Friendship Hospital, Capital Medical University, Beijing 100050, China; foxtail_09@hotmail.com (X.-C.F.); malinthe@yeah.net (L.-S.M.); 2 Department of Orthodontics, Beijing Stomatological Hospital & School of Stomatology, Capital Medical University, Beijing 100050, China; lydiach323@ccmu.edu.cn; 3 Clinical Research Center, Division of Conservative Dentistry and Periodontology, School of Dentistry, Medical University of Vienna, 1090 Vienna, Austria; dentistdiwakarsingh@gmail.com

**Keywords:** temporomandibular joint, cone-beam computed tomography, malocclusions, articular eminence inclination

## Abstract

(1) Background—The aim of the present study was to evaluate the correlation between the temporomandibular joint (TMJ) osseous morphology of normal skeletal pattern individuals with different dental malocclusions by using cone-beam computed tomography (CBCT). (2) Methods—The CBCT images of bilateral TMJs in 67 subjects with skeletal class I and average mandibular angle (26 males and 41 females, age range 20–49 years) were evaluated in this study. The subjects were divided into class I, class II division 1, and class II division 2 according to the molar relationship and retroclination of the maxillary incisors. Angular and linear measurements of TMJ were evaluated and the differences between the groups were statistically analyzed. (3) Results—Intragroup comparisons showed statistical differences for articular eminence inclination, the width of the glenoid fossa, the ratio of the width of the glenoid fossa to the depth of the glenoid fossa, the condylar angle, and the intercondylar angle between the malocclusion groups. The measurements of the glenoid fossa shape showed no significant difference between the left and right sides. Females showed more differences in the morphological parameters of TMJ between the three malocclusion groups than the males. (4) Conclusion—The present study revealed differences in the TMJ osseous morphology between dental class I and class II malocclusions in the normal skeletal pattern.

## 1. Introduction

The temporomandibular joint (TMJ) is one of the most complex joints in the human body. It is formed by the condyle of the mandibular, the inferior component of the joint, and the glenoid fossa forming the superior component of the joint, which is located at the inferior aspect of the squamous part of the temporal bone [1,2]. The joint cavity is separated into the upper and lower compartments by the articular disk, which is made of avascular and aneural dense fibrous connective tissue [3]. The unique anatomy of the TMJ allows for the hinging movement of the mandible and is therefore considered a ginglymoid joint. It can also provide gliding movements and is therefore also an arthrodial joint; thus, it is technically considered a compound joint.

Form and function are considered to be closely linked, and it follows that the osseous morphology of the TMJ might be related to the dynamic balance of mandibular functions in three dimensions. During the mandibular movement, the condyle-disk complex process slides over the posterior slope of the articular eminence. The inclination of articular eminence dictates the path of condylar movement, as well as the degree of rotation of the articular disk over the condyle [4,5]. For patients with a steeper articular eminence, the condyle is forced to move more inferior and the disk rotates more prominent when protruding or opening. This may lead the mandible to move more vertically during the functional movement [6]. It is reported that a patient with steeper articular eminence is more likely to develop internal dysfunctions, such as anterior disk displacements, than a patient with a flatter articular eminence [7,8].

The articular eminence is sometimes described as the anterior limit of the glenoid fossa. The quantitative evaluation of the articular eminence morphology can be assessed using the inclination, length, and height, where the articular eminence inclination (AEI) is defined as the angle formed by the articular eminence and the horizontal reference plane, which may be the Frankfort horizontal (FH) plane, the true horizontal line, the anterior nasal spine to the posterior nasal plane (ANS–PNS), or the occlusal plane [4,9,10,11]. The normal value of the AEI in adults has been reported to be 30 to 60 degrees. This angle is not only related to physiological factors, such as age, gender, tooth inclination, dental arch morphology, and the facial growth pattern [4,12,13,14], but also pathological factors, including occlusion change, TMJ osteoarthritis, internal derangements, and posterior tooth loss [15,16,17,18].

A thorough understanding of the morphology and anatomical features of the TMJ is crucial such that we can distinguish the normal condition from the abnormal variant. It is reported that the surface of the structural features of the glenoid fossa may take part in remodeling and reconfiguring following the mechanical and functional conditions to which the adjacent structures are subjected [19]. Some authors suggested that changing the relationship between the upper and lower dentition may lead to right-to-left-side differences in masticatory muscles, which affect the relative relationship of the condyle and glenoid fossa [20,21]. The effect of occlusal factors on the morphology of the temporomandibular joint remains to be clarified. Based on this context, we hypothesized that the discrepancy of the occlusion relationship may be an independent factor that affects the morphology of the TMJ. Thus, the purpose of the present study was to evaluate the correlation between the TMJ osseous morphology of the normal skeletal pattern individuals with different dental malocclusion by using cone-beam computed tomography (CBCT).

## 2. Materials and Methods

### 2.1. Data Collection and Grouping

The present study was performed at the Department of Stomatology, Beijing Friendship Hospital, Capital Medical University, and it was approved by the Ethical Committee of Beijing Friendship Hospital (approval number 2021-P2-008-01, updated on 1 February 2021). High-resolution CBCT imaging volumes were obtained from examinations that were previously conducted for orthodontic purposes between January 2019 to December 2020; therefore, they had no connection to the present study.

The age of the patients in the sample selected for the study needed to be no less than 20 years old. The sagittal skeletal relationship was defined using the ANB angle (ANB), Frankfurt horizontal–mandibular plane angle (FH–MP), and sella–nasion to gnathion–gonion angle (SN–GnGo), which were measured from the lateral cephalograms that were automatically reconstructed and generated using the QR-NNT Viewer version 5.6 software program (Quantitative Radiology, Verona, Italy). The participants included were limited to skeletal class I with an average mandibular angle, which was defined as 0.7° ≤ ANB ≤ 4.7°, 21.2° ≤ FH–MP ≤ 33.4°, 27.3° ≤ SN–GnGo ≤ 37.7° [22]. The exclusion criteria were as follows: (1) evidence of temporomandibular disorders (TMDs) in a clinical or imaging examination; (2) previous history of orthodontics or TMJ treatment; (3) craniofacial syndrome or anomalies, such as cleft lip and palate; (4) systemic diseases, such as rheumatic arthritis and rheumatoid arthritis; (5) deciduous or missing teeth, except third molars; (6) asymmetric molar relationship or class III molar relationship from the plaster models before treatment; (7) fracture or other pathologies in the region of the TMJs, such as anomalies, tumors, ankylosis, or degenerative changes; (8) poor image quality. After applying the inclusion and exclusion criteria, 67 patients (26 males and 41 females, age range 20–49 years) were included and recorded bilaterally; in total, 134 TMJs were evaluated.

The included samples were divided into three groups: class I (CI), class II division 1 (CII-1), and class II division 2 (CII-2). The assignment to each group was done based on the molar relationship on the patient’s plaster models before treatment, and the class II groups were then divided according to the retroclination of the maxillary incisors. The interval between the model making and CBCT taking should be less than 1 week. Each study group was then subdivided according to gender and left or right side.

### 2.2. Simple Size Calculation

Based on our preliminary data, we got a minimum detectable difference value of 5, and we calculated the sample size using the Power Analysis and Sample Size software version 11.0 (PASS, NSCC, LLC, Kaysville, Utah, USA) in the present study. Considering a study with a two-tailed hypothesis, for an α value of 0.05, a β value of 0.2, and a statistical power of 80%, the minimum sample size was computed to be 38 subjects per group.

### 2.3. Acquisition of CBCT Images

The CBCT scans were performed using a New Tom 5G version FP (Quantitative Radiology, Verona, Italy) flat-panel-based CBCT machine with a field of view of 18 × 16 cm. The scanner operated with a maximum output of 110 kV and 5 mAs, exposure time of 3.6 s, and a voxel size of 0.15 mm. The patients with teeth in the maximum intercuspation position were placed in a horizontal position according to the laser indicators and we ensured that the Frankfort horizontal plane was perpendicular to the flat panel of the device in order to obtain a consistent orientation of sagittal images. All CBCT scans were obtained under the same scanning conditions by the same experienced oral radiologist with the same device.

### 2.4. Measurements

The CBCT examination results were analyzed using the QR-NNT Viewer version 5.6 software program, which was the proprietary software of the New Tom 5G CBCT system. Before the quantitative evaluation, a secondary calibration was performed to ensure the Frankfort plane was held parallel to the horizontal plane on the sagittal reference view. The CBCT data were also spatially oriented by aligning the anterior and posterior nasal spine on the axial reference view. Digital reconstruction was then conducted in the TMJ regions.

**Figure 1:**
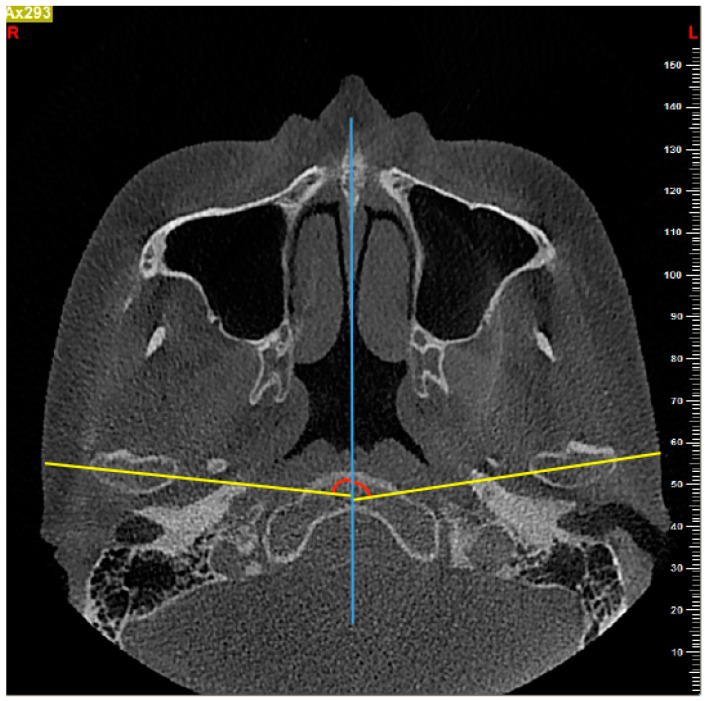
The measurement of condylar angle (CA) on the axial slice. The angle between the long axis of the condyle (yellow line) and the midsagittal plane (blue line) was measured.

**Figure 2:**
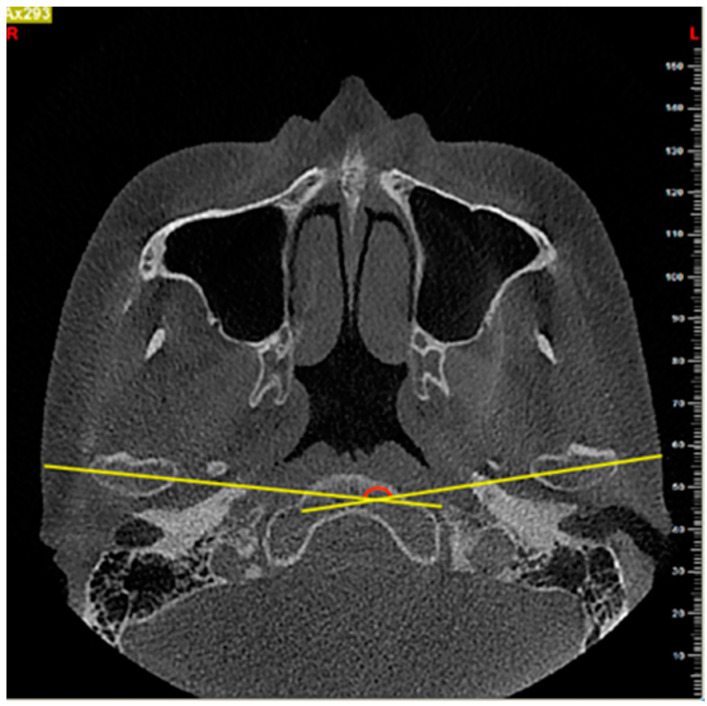
The measurement of intercondylar angle (IA) on the axial slice. The angle between the long axis of the left and right condyle (yellow line) was measured.

On the axial view, the slice of the condylar processes that had the widest mediolateral extent on both sides of TMJ was used to measure the angulation of the condyles, which involved two variables:

(1) Condylar angle (CA): the angle between the long axis (the line passing through the medial and lateral pole of the condyle) of the left or right condyle and the midsagittal plane in the axial view (Figure 1).

(2) Intercondylar angle (IA): the angle between the long axis of the right and left condyles in the axial view (Figure 2).

**Figure 3:**
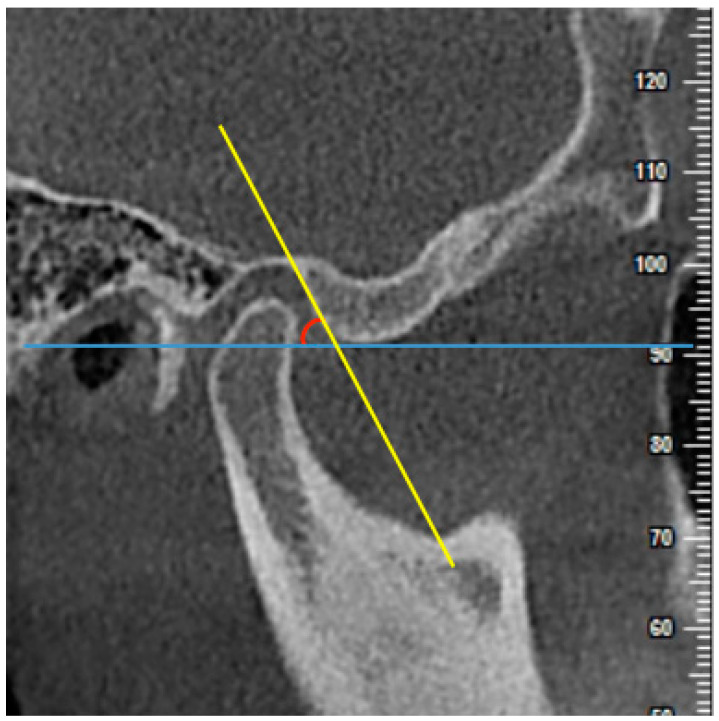
The measurement of articular eminence inclination (AEI) using the best-fit line method (AEI-BFL) on the central sagittal slice. The angle between the tangent line drawn to the posterior slope of the articular eminence (yellow line) and a line parallel to the Frankfort horizontal (FH) plane (blue line) was measured.

**Figure 4:**
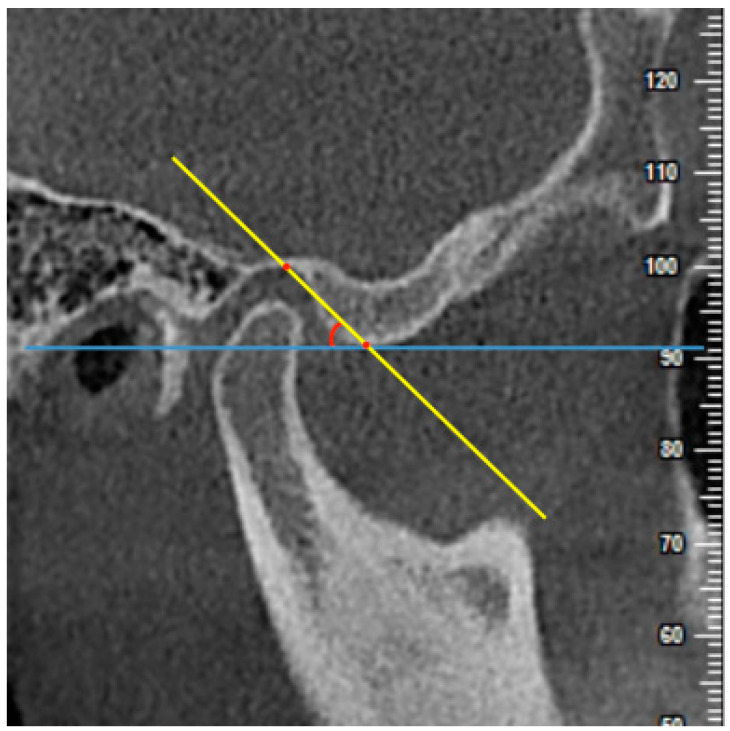
The measurement of AEI using the top-roof line method (AEI-TRL) on the central sagittal slice. The angle between the “top-roof line (the line connecting the crest point of the articular eminence and the roof of the glenoid fossa)” (yellow line) and a line parallel to the FH plane (blue line) was measured.

**Figure 5:**
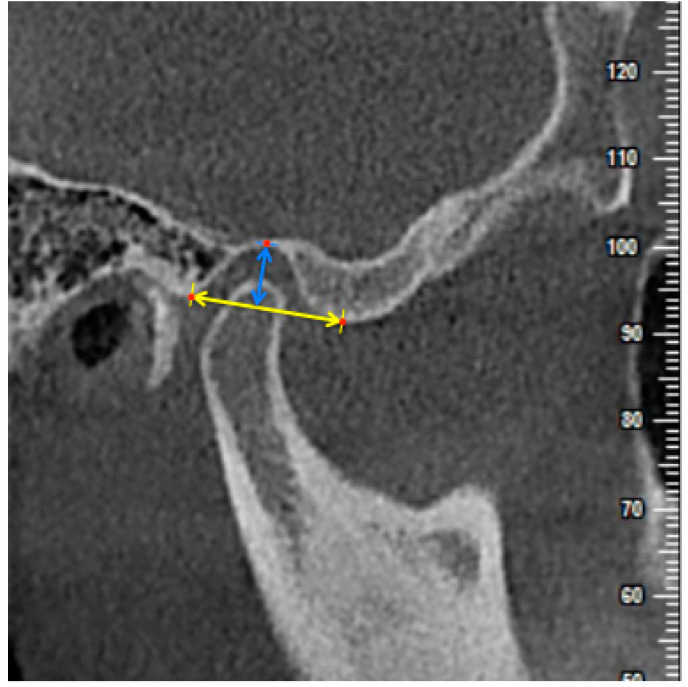
The measurement of the width of the glenoid fossa (GFW) and depth of the glenoid fossa (GFD) on the central sagittal slice. The distance between the crest point of the articular eminence and the posterior part of the glenoid process was measured as the GFW (yellow arrow) and the perpendicular distance between the highest point of the glenoid fossa and the GFW line was measured as the GFD (blue arrow).

**Figure 6:**
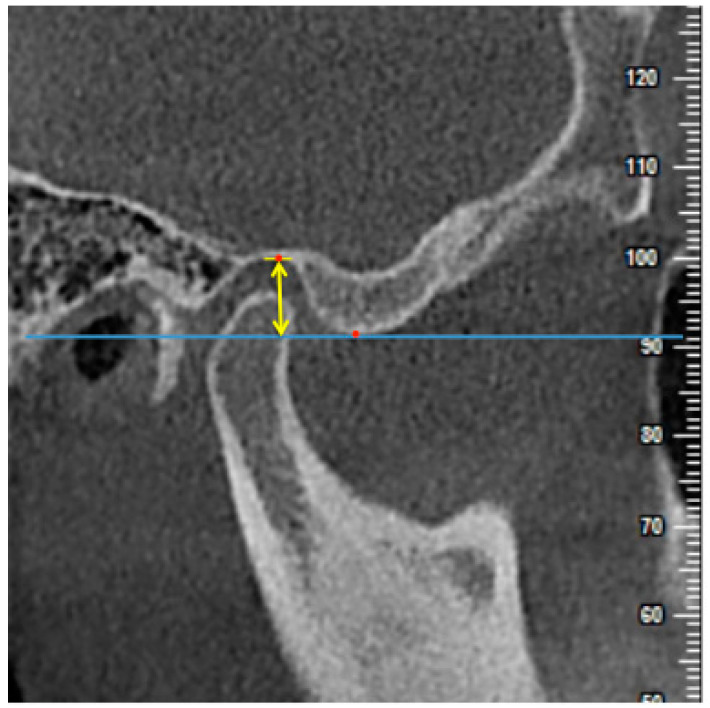
The measurement of the height of the articular eminence (AEH) on the central sagittal slice. The perpendicular distance between the line parallel to the FH plane through the crest point of the articular eminence (blue line) and the highest point of the glenoid fossa was measured (yellow arrow).

The chosen slice was also used as the reference view for the secondary reconstruction of the sagittal slices [13], on which a line parallel to the long axis of the condylar process was drawn and sagittal images were reconstructed with a 0.5 mm slice interval and a 0.5 mm thickness. The following variables were calculated on the central sagittal slice of the TMJ:

(1) AEI using the best-fit line method (AEI-BFL): the angle between the tangent line drawn to the posterior slope of the articular eminence and a line parallel to the FH plane (Figure 3).

(2) AEI using the top-roof line method (AEI-TRL): the angle between the “top-roof line” of the articular eminence (the line connecting the crest point of the articular eminence and the roof of the glenoid fossa) and a line parallel to the FH plane (Figure 4).

(3) Width of the glenoid fossa (GFW): the distance between the crest point of the articular eminence and the posterior part of the glenoid process.

(4) Depth of the glenoid fossa (GFD): the perpendicular distance between the highest point of the glenoid fossa and the GFW line (the line passing through the crest point of the articular eminence and the posterior part of the glenoid process) (Figure 5).

(5) Ratio of the GFW to the GFD (GFW/GFD).

(6) Height of the articular eminence (AEH): the perpendicular distance between the highest point of the glenoid fossa and the line parallel to the FH plane through the crest point of the articular eminence (Figure 6).

The measurements and angles evaluated on both the axial and central sagittal slices were obtained according to the methods mentioned by İlgüy, Park, Sümbüllü, and Paknahad [13,23,24,25]. All the assessments were performed independently by two operators (X.-C.F. and L.-S.M.) and the mean of the results was used for the statistical analysis.

### 2.5. Measurements Precision

To test the reliability of the measurements, 30 joints (10 joints from each group) were randomly selected from the collected samples and measured twice with a 1-week interval by the same operators (X.-C.F. and L.-S.M.). The first and the second series of measurements were compared using a paired *t*-test to check for systematic error at a significance level of *p* < 0.05. The random errors were assessed using the intraclass correlation coefficient (ICC) [26].

### 2.6. Statistical Analysis

All the variables were analyzed using the Statistical Package for Social Sciences software version 20.0 (SPSS, IBM, New York, NY, USA). The one-way analysis of variance (ANOVA) followed by the Bonferroni multiple comparisons test was used to analyze the statistical differences between three malocclusion groups. A paired *t*-test and an independent sample *t*-test were applied to determine the possible differences between the left–right sides and the genders in the same malocclusion group, respectively. A *p*-value < 0.05 was considered statistically significant.

## 3. Results

### 3.1. Error of the Study

The paired *t*-test showed no statistically significant differences between the data obtained from the different operators and double measurements from the same operator at a significant level of 0.05. The ICC for intra-operators (operator 1: *r* = 0.981–0.987; operator 2: *r* = 0.875–0.912) and inter-operators (*r* = 0.871–0.901) showed excellent agreement and good reliability for all the measures analyzed.

### 3.2. Descriptive Statistics of Age and Basic Measurements of the Skeletal Pattern

A total of 67 high-resolution CBCT imaging volumes with skeletal class I (mean ANB angle of 3.44 ± 1.05°) and average mandibular angle (mean FH–MP of 26.52 ± 3.76° with a mean SN–GnGo of 33.22 ± 3.35°) were collected. The mean age of the participants of the present study was 27.91 ± 6.94 years. The means and standard deviations for age and the angular measurements of the skeletal pattern for the different malocclusion groups are presented in Table 1. The intergroup results showed that there were no statistically significant differences between the three malocclusion groups.

### 3.3. Measurements of the Temporomandibular Joint According to Malocclusion

The distributions of the TMJ osseous morphology measurements in the three malocclusion groups are summarized in Table 2. By using the one-way ANOVA, all the angular and linear measurements showed significant differences between the three groups, except for the GFD and AEH (*p* < 0.05). The Bonferroni multiple comparisons test further showed that the AEI found using the best-fit line method of class II division 2 was significantly higher than the class II division 1 (*p* = 0.017), followed by the class I AEI (*p* = 0.000). However, the difference was not obvious between the class II division 1 and class II division 2 (*p* = 1.000) for the AEI found using the top-roof methods. The widths of the glenoid fossa of the three groups were 17.37 ± 1.60 mm (C-I), 16.86 ± 1.40 mm (CII-1), and 16.59 ± 1.28 mm (CII-2). The indicators of the GFW and GFW/GFD only presented differences between the class I and the class II division 2 groups. As for the measurements of the condyle on the axial slice, the condylar and intercondylar angles of the class II division 2 group were lower than the other two groups (Table 3).

### 3.4. Descriptive Statistics of the Measurements of the TMJ According to the Left and Right Side

Table 4 lists the mean values and standard deviations of the TMJ morphology measurements for the left and right sides of the three malocclusion groups. According to the paired *t*-test, only the variables of GFW/GFD and CA in the class II division 1 group and CA in the class II division 2 group showed significant differences (*p* < 0.05).

### 3.5. Descriptive Statistics of the Measurements of the TMJ According to Gender

The differences between the male and female participants of the same occlusion pattern are shown in Table 5. No statistically significant differences were observed between both genders among three malocclusion groups except for the two angular variables of the condyle in the class I group and the GFW/GFD ratio of the class II division 1 group.

A comparison of the three malocclusion groups according to gender using one-way ANOVA followed by the Bonferroni multiple comparisons is illustrated in Table 6. The AEI evaluated using two methods presented significant differences between different malocclusion groups in both genders (*p* < 0.05). In addition, the indicators of GFW, GFW/GFD, CA, and IA showed more intergroup differences in females than in males.

## 4. Discussion

TMJ is a region with high anatomical complexity, whereas the clinical examination can only provide us with very limited information because it is hard to precisely reveal the internal environment. Taking this restriction into consideration, various radiographic methods were selected to evaluate the morphology of the TMJ in previous studies. Conventional two-dimensional radiographs, such as tomography or panoramic radiographs, were widely used in the early days. However, these modalities are inadequate for quantitative evaluation because of certain limitations, for example, they cannot reflect the three-dimensional shape accurately and may have image distortion and magnification [1,27]. Magnetic resonance imaging (MRI) can provide visualization in both osseous and soft tissue abnormalities, including the morphology of bone structures, the articular disk, and associated muscles and ligaments, in addition to evaluating the functional relationships between them [28]. It is considered the gold standard imaging diagnostic method for TMDs and is widely used in the qualitative evaluation of TMDs [28]. Unfortunately, it was difficult for us to use MRI for all participants included in the present study due to the limitations of the research conditions. The appearance of helical CT makes it possible to evaluate osseous components in three dimensions without superimposition or distortion. Nowadays, CBCT has already replaced helical CT as a superior method in the stomatological area because of the high spatial resolution, lower radiation dose, shorter scanning time, and greater cost-effectiveness [24,25]. In this study, the CBCT was selected for angular and linear measurements of the TMJ osseous morphology.

The development stage of the articular eminence may influence the quantitative measurements of the TMJ. After reviewing the previous studies, the time to full development time of the articular eminence is still controversial. An autopsy study of Oberg reported that the tubercle and the fossa were well developed at the age of 14–15 years [29]. On the other hand, Katsavrias studied the dry skulls from Asiatic Indian individuals in 2002 and found that the articular eminence was 90–94% complete by the age of 20 years [4]. In order to minimize the influence of the growth on the experimental result, we limited the age of the patients in the sample selection to those that were at least 20 years old. Finally, the age range of the samples included in the present study was 20–49 years and the mean age was 27.91 ± 6.94 years. The sample size for understanding anatomical trends in patients should be as large as possible; however, the present study was just a pilot investigation that demonstrated the possibility of a trend existing. We calculated the sample size using the PASS software based on our preliminary data to increase the scientificity of the study, where the minimum sample size was computed to be 38 joints per group. It should be recognized that the present study aimed to access the association between the osseous morphology of the TMJ and the dental malocclusion. Therefore, the skeletal pattern of the individuals of the current study was strictly limited to skeletal class I with average mandibular angle by ANB, FH–MP, and SN–GnGo. After the statistical analysis of the age and basic measurement of the skeletal pattern, there was no statistical difference between the different malocclusion groups, which indicated that the samples of different malocclusion groups had excellent intergroup consistency for comparison.

The articular eminence is a small bone structure belonging to the cranium. The surface of its posterior slope is exposed to mechanical and functional load arising from biomechanical forces from other structures within the TMJ, where these loads influence the morphological characteristics of it [30]. It is crucial to choose a stable and comparable method for measuring the inclination of the articular eminence. The “best-fit line” method and the “top-roof line” method on the central sagittal slice of the TMJ are the two main methods described in previous studies [13]. The “best-fit line” method is considered as the functional inclination of the articular eminence because it is directly related to the movement direction of the condyle–disk complex and reflects the actual condylar path. In contrast, the “top-roof line” method is more concerned about the localization of the articular eminence in relation to the glenoid fossa and it largely depends on the development of the articular eminence. Therefore, it depicts the anatomical inclination of the articular eminence better. In the current study, the class II division 2 group showed the highest value of AEI-BFL, followed by the class II division 1 group, then the class I group, where the differences between the three groups were significant. For the AEI-TRL, class II division 2 also revealed the highest value. However, the statistical differences were only found between the dental class I and class II malocclusions. These results indicated that there might be some correlation between the AEI-TRL and the molar relationship. However, for the functional AEI, the angle was not only related to the molar relationship but was also affected by the inclination of the anterior teeth.

In previous studies, the fossa shapes were assessed in subjective ways and traditionally classified as triangular, trapezoidal, oval, and round [31]. In this study, the shapes of the fossa were studied quantitatively using their width and depth. Considering that the size of the fossa may have great variability in different individuals, we also introduced the variable of GFW/GFD to describe the relative relationship between the width and depth. The GFD and AEH were both used to analyze the vertical depth of the fossa; however, the GFD is focused more on describing the anatomical height of the glenoid fossa, regardless of the patient’s head position. The AEH was highly related to the shape of the articular eminence, which reflected the vertical sliding space of the condyle in the normal head position. Based on the results of this study, the difference in the fossa shapes only appeared in the GFW and its ratio to GFD between the class II division 2 and class I groups. There were no significant differences in the anatomic and functional fossa depths between different malocclusion groups. It indicated that the fossa shapes of class II divisions 1 and 2 were relatively similar, which was consistent with the findings obtained by Katsavrias and Halazonetis [32]. In addition, the height of the articular fossa might not be a specific index to distinguish between different malocclusions according to samples of the study. Moreover, Sümbüllü et al., Cğlayan et al., and Poluha et al. [24,33,34] affirmed that the AEH and GFD were also not specific indicators to discriminate between the normal and TMD patients, though the opposite opinion was expressed by Paknahad et al. [25].

TMJ is the only diarthrodial joint with a bilateral linkage in human bodies. It can move synchronously during the symmetrical movement (open–close, protrusion–retrusion) or with its own movements on each side during the lateral movement. Several published papers only noted TMJ as an individual joint without taking into account the contralateral side [24,25]. In the present study, the left and right joints were measured separately and the differences between both sides were evaluated. Based on the results of the study, all the angular and linear measurements of the glenoid fossa showed no significant differences between the left and right sides. The findings of Shahidi et al. and Wu et al. also mentioned that the inclination of the left and right articular eminences did not display any significant differences, which is in agreement with the current study [1,11]. However, the condylar angle of the left joint in both class II division 1 and division 2 groups was significantly lower than that of the right, which was not seen in the class I group. This may indicate that the mandible of the class II patients revealed more asymmetry than that of the class I patients. The values of CA and IA also showed differences between different malocclusion groups. Compared with other types of malocclusion, the condyles of individuals in the class II division 2 group had a greater tendency to rotate inward.

The morphological discrepancies of TMJ due to differences in sex hormones and metabolic activity between male and female individuals have been reported in previous studies [35]. Beyond that, differences in the functional loading of TMJ according to gender can also cause changes in TMJ morphology [36]. Jasinevicius et al. [37] found a gender difference in AEI, which demonstrated a contrary result to the study of Sümbüllü et al. [24]. Based on our results, it was observed that the diversities of TMJ morphology between the two genders were only revealed in the CA and IA values of the class I group. As for the differences in the TMJ morphology variables between malocclusion groups that were separately analyzed according to gender, the AEI showed similar trends in different genders. However, the differences in other morphological parameters of both the glenoid fossa and condyle in female individuals between the three malocclusion groups mentioned in the current study were higher than those in males, which might be one of the possible reasons why TMJ dysfunctions occur more often in females than in males.

## 5. Conclusions

On the basis of our study, the following conclusions could be drawn:

1. The inclination of articular eminence displayed a great difference between class I and class II malocclusions in the normal skeletal pattern, and the individuals of class II division 2 showed the highest AEI.

2. The height of the glenoid fossa might not be a specific index to distinguish between different malocclusions.

3. The condyles of individuals in the class II division 2 group had a greater tendency to rotate inward.

4. The shape of the glenoid fossa showed no significant difference between the left and right sides.

5. The differences in morphological parameters of TMJ in female individuals between the three malocclusion groups were higher than those in males.

## Figures and Tables

**Table 1:** Descriptive statistics of age and the basic measurements of the skeletal patterns.

Variable	Total (*N* = 67)		Class I (*n* = 24)		Class II-1 (*n* = 20)		Class II-2 (*n* = 23)		*F*-Value	*p*-Value
	Mean ± SD	Range	Mean ± SD	Range	Mean ± SD	Range	Mean ± SD	Range		
Age (year)	27.91 ± 6.94	20–49	27.00 ± 5.56	20–40	27.50 ± 8.52	20–49	29.22 ± 6.84	20–45	0.642	0.530
ANB (°)	3.44 ± 1.05	1.1–4.9	3.10 ± 1.07	1.1–4.6	3.41 ± 0.97	1.4–4.7	3.84 ± 1.02	1.6–4.9	3.125	0.051
FH–MP (°)	26.52 ± 3.76	21.2–33.3	26.58 ± 4.15	21.2–33.2	26.43 ± 3.12	22.0–33.1	26.53 ± 4.00	21.2–33.3	0.009	0.991
SN–GnGo (°)	33.22 ± 3.35	27.4–37.7	33.06 ± 3.43	27.4–37.6	33.43 ± 2.88	28.3–37.6	33.21 ± 3.75	27.4–37.7	0.066	0.937

ANB: ANB angle; FH–MP: Frankfurt horizontal–mandibular plane angle; SN–GnGo: sella-nasion to gnathion-gonion angle.

**Table 2:** Measurements of the temporomandibular joint osseous morphology according to the malocclusion.

Variable	Class I			Class II-1			Class II-2			*F*-Value	*p*-Value
	*n*	Mean ± SD	Range	*n*	Mean ± SD	Range	*n*	Mean ± SD	Range		
AEI-BFL (°)	48	52.56 ± 7.01	39.7–68.7	40	62.06 ± 5.85	49.1–75.6	46	66.43 ± 8.30	47.5–82.7	45.799	0.000 **
AEI-TRL (°)	48	38.16 ± 5.43	29.4–48.4	40	42.21 ± 5.45	32.5–55.7	46	42.84 ± 4.86	32.7–52.8	10.929	0.000 **
GFW (mm)	48	17.37 ± 1.60	13.8–21.0	40	16.86 ± 1.40	14.8–19.7	46	16.59 ± 1.28	14.1–19.3	3.615	0.030 *
GFD (mm)	48	5.92 ± 1.08	3.9–8.4	40	6.09 ± 0.95	3.7–8.1	46	6.40 ± 0.96	3.9–8.5	2.760	0.067
GFW/GFD	48	3.01 ± 0.49	2.14–4.19	40	2.83 ± 0.47	2.09–4.03	46	2.63 ± 0.33	2.16–3.67	8.843	0.000 **
AEH (mm)	48	7.15 ± 1.30	5.1–10.4	40	7.53 ± 0.93	5.0–10.2	46	7.45 ± 1.22	4.8–10.3	1.270	0.284
CA (°)	48	73.94 ± 5.71	59.8–85.5	40	73.64 ± 7.17	55.8–86.1	46	69.87 ± 6.31	58.0–84.1	5.775	0.004 **
IA (°)	24	147.87 ± 10.46	125.9–168.1	20	147.29 ± 13.29	114.4–165.4	23	139.01 ± 12.03	114.4–168.1	3.96	0.024 *

AEI-BFL: AEI found using the best-fit line method; AEI-TRL: AEI found using the top-roof line method; GFW: width of the glenoid fossa; GFD: depth of the glenoid fossa; GFW/GFD: ratio of the GFW to the GFD; AEH: height of the articular eminence; CA: condylar angle; IA: intercondylar angle; *: *p*-value < 0.05; **: *p*-value < 0.01.

**Table 3:** Bonferroni test results for the measurements of the temporomandibular joint for the three malocclusion groups.

Variable	CI to CII-1		CI to CII-2		CII-1 to CII-2	
	Mean Difference (I–J)	*p*-Value	Mean Difference (I–J)	*p*-Value	Mean Difference (I–J)	*p*-Value
AEI-BFL	−9.4975	0.000 **	−13.8658	0.000 **	−4.3683	0.017 *
AEI-TRL	−4.0496	0.001 **	−4.6831	0.000 **	−0.6335	1.000
GFW	0.5138	0.289	0.7818	0.028 *	0.2680	1.000
GFD	−0.01692	1.000	−0.4813	0.065	−0.3122	0.458
GFW/GFD	0.18000	0.163	0.37549	0.000 **	0.19549	0.116
AEH	−0.3729	0.421	−0.2936	1.000	0.0793	1.000
CA	0.2929	1.000	4.0680	0.007 **	3.7751	0.021 *
IA	0.58583	1.000	8.86214	0.039 *	8.27630	0.079

CI: class I; CII-1: class II division 1; CII-2: class II division 2; AEI-BFL: AEI found using the best-fit line method; AEI-TRL: AEI found using the top-roof line method; GFW: width of the glenoid fossa; GFD: depth of the glenoid fossa; GFW/GFD: ratio of the GFW to the GFD; AEH: height of the articular eminence; CA: condylar angle; IA: intercondylar angle; *: *p*-value < 0.05; **: *p*-value < 0.01.

**Table 4:** Descriptive statistics of the measurements of the temporomandibular joint according to the left and right sides for the three malocclusion groups.

Variable	Class I (*n* = 24)			Class II-1 (*n* = 20)			Class II-2 (*n* = 23)		
	Left Side	Right Side	*p*-Value	Left Side	Right Side	*p*-Value	Left Side	Right Side	*p*-Value
AEI-BFL (°)	52.78 ± 6.27	52.34 ± 7.83	0.710	62.95 ± 6.50	61.17 ± 5.14	0.246	65.82 ± 7.44	67.04 ± 9.22	0.346
AEI-TRL (°)	38.23 ± 6.05	38.09 ± 4.86	0.821	43.29 ± 6.22	41.14 ± 4.46	0.098	42.97 ± 4.29	42.72 ± 5.47	0.739
GFW (mm)	17.21 ± 1.68	17.53 ± 1.53	0.232	16.95 ± 1.45	16.76 ± 1.37	0.337	16.56 ± 1.25	16.61 ± 1.33	0.824
GFD (mm)	5.74 ± 1.01	6.10 ± 1.15	0.084	5.92 ± 1.05	6.27 ± 0.83	0.099	6.34 ± 1.07	6.47 ± 0.87	0.518
GFW/GFD	3.07 ± 0.50	2.95 ± 0.48	0.128	2.94 ± 0.48	2.72 ± 0.43	0.024 *	2.67 ± 0.37	2.60 ± 0.28	0.393
AEH (mm)	7.22 ± 1.34	7.09 ± 1.29	0.616	7.67 ± 0.96	7.38 ± 0.89	0.234	7.48 ± 1.14	7.41 ± 1.32	0.725
CA (°)	73.09 ± 5.64	74.78 ± 5.77	0.083	72.08 ± 7.20	75.21 ± 6.96	0.010 **	68.07 ± 5.93	71.67 ± 6.28	0.001 **

AEI-BFL: AEI found using the best-fit line method; AEI-TRL: AEI found using the top-roof line method; GFW: width of the glenoid fossa; GFD: depth of the glenoid fossa; GFW/GFD: ratio of the GFW to the GFD; AEH: height of the articular eminence; CA: condylar angle; IA: intercondylar angle; *: *p*-value < 0.05; **: *p*-value < 0.01

**Table 5:** Descriptive statistics of the measurements of the temporomandibular joint according to gender among the three malocclusion groups.

Variable	Class I			Class II-1			Class II-2		
	Male (*n* = 22/11)	Female (*n* = 26/13)	*p*-Value	Male (*n* = 14/7)	Female (*n* = 26/13)	*p*-Value	Male (*n* = 16/8)	Female (*n* = 30/15)	*p*-Value
AEI-BFL (°)	53.14 ± 5.90	52.08 ± 7.92	0.608	60.95 ± 5.24	62.66 ± 6.17	0.386	64.40 ± 5.21	67.51 ± 9.46	0.158
AEI-TRL (°)	37.96 ± 4.67	38.33 ± 6.08	0.816	43.21 ± 6.19	41.67 ± 5.05	0.399	42.45 ± 4.31	43.05 ± 5.19	0.693
GFW (mm)	17.26 ± 1.61	17.46 ± 1.61	0.679	16.36 ± 1.27	17.12 ± 1.41	0.099	16.73 ± 1.33	16.51 ± 1.26	0.597
GFD (mm)	6.14 ± 1.07	5.74 ± 1.08	0.209	6.36 ± 1.04	5.94 ± 0.88	0.183	6.69 ± 0.84	6.25 ± 1.00	0.135
GFW/GFD	2.87 ± 0.38	3.13 ± 0.55	0.070	2.62 ± 0.35	2.94 ± 0.48	0.032 *	2.52 ± 0.23	2.69 ± 0.35	0.081
AEH (mm)	7.30 ± 1.39	7.03 ± 1.24	0.489	7.40 ± 0.67	7.59 ± 1.04	0.538	7.59 ± 1.14	7.37 ± 1.27	0.554
CA (°)	70.64 ± 5.53	76.72 ± 4.24	0.000 **	72.07 ± 5.99	74.49 ± 7.70	0.315	69.49 ± 6.65	70.07 ± 6.22	0.769
IA (°)	141.28 ± 9.89	153.45 ± 7.38	0.002 **	144.14 ± 8.12	148.98 ± 15.43	0.453	136.9 ± 13.19	140.13 ± 11.69	0.552

AEI-BFL: AEI found using the best-fit line method; AEI-TRL: AEI found using the top-roof line method; GFW: width of the glenoid fossa; GFD: depth of the glenoid fossa; GFW/GFD: ratio of the GFW to the GFD; AEH: height of the articular eminence; CA: condylar angle; IA: intercondylar angle; *: *p*-value < 0.05; **: *p*-value < 0.01.

**Table 6:** Statistical summary of the measurements of the temporomandibular joint according to malocclusion in different genders.

Variable	Male					Female		
	*F*-Value	*p*-Value			*F*-Value	*p*-Value		
		CI to CII-1	CI to CII-2	CII-1 to CII-2		CI to CII-1	CI to CII-2	CII-1 to CII-2
AEI-BFL (°)	20.840 **	0.000 **	0.000 **	0.238	26.337 **	0.000 **	0.000 **	0.082
AEI-TRL (°)	6.001 **	0.011 *	0.027 *	1.000	5.430 **	0.090	0.005 **	1.000
GFW (mm)	1.784	0.216	0.783	1.000	3.178 *	1.000	0.047 *	0.344
GFD (mm)	1.444	1.000	0.287	1.000	1.872	1.000	0.178	0.766
GFW/GFD	5.549 **	0.096	0.008 **	1.000	6.177 **	0.473	0.002 **	0.144
AEH (mm)	0.307	1.000	1.000	1.000	1.459	0.281	0.890	1.000
CA (°)	0.689	1.000	1.000	0.739	8.376 **	0.597	0.000 **	0.029 *
IA (°)	0.897	1.000	1.000	0.602	4.546 *	1.000	0.017 *	0.174

CI: class I; CII-1: class II division 1; CII-2: class II division 2; AEI-BFL: AEI found using the best-fit line method; AEI-TRL: AEI found using the top-roof line method; GFW: width of the glenoid fossa; GFD: depth of the glenoid fossa; GFW/GFD: ratio of the GFW to the GFD; AEH: height of the articular eminence; CA: condylar angle; IA: intercondylar angle; *: *p*-value < 0.05; **: *p*-value < 0.01.

## Data Availability

The data presented in this study are available on request from the corresponding author.
